# Silencing the second harmonic generation from plasmonic nanodimers: A comprehensive discussion

**DOI:** 10.3762/bjnano.9.250

**Published:** 2018-10-15

**Authors:** Jérémy Butet, Gabriel D Bernasconi, Olivier J F Martin

**Affiliations:** 1 Nanophotonics and Metrology Laboratory (NAM), Swiss Federal Institute of Technology Lausanne (EPFL), 1015 Lausanne, Switzerland

**Keywords:** gold, nanoantennas, nonlinear plasmonics, second harmonic generation, surface integral equation method

## Abstract

The silencing of the second harmonic generation process from plasmonic nanostructures corresponds to the limited far-field second harmonic radiation despite the huge fundamental electric field enhancement in the interstice between two plasmonic nanoparticles forming a nanodimer. In this article, we report a comprehensive investigation of this effect using a surface integral equation method. Various geometries are considered, including nanoantennas with cylindrical and rectangular arms as well as nanodimers with surface defects. The existence of the silencing of the second harmonic generation from plasmonic nanogaps is first confirmed, and the problem of the origin of the second harmonic light from these plasmonic nanostructures is addressed in detail. Our results show that the distribution of the second harmonic sources, especially on the arm sides, plays a non-negligible role in the overall second harmonic emission. This contribution is induced by retardation effects at the pump wavelength and results in a dipolar second harmonic emission.

## Introduction

Due to their high density of free electrons, plasmonic nanostructures offer the possibility to concentrate light into subwavelength regions [1–2]. The collective oscillations of these electrons in a given plasmonic nanostructure are called localized surface plasmon resonances (LSPRs) [3–5]. The high electric field enhancement associated with the optical excitation of such a resonance has been proven to be an important, practical way to control light–matter interaction down to the nanoscale [3–5]. To even further enhance this interaction, it was proposed to couple two plasmonic nanostructures by bringing them close to each other, resulting in a nanoantenna made of two arms separated by a gap of a few nanometers [6]. Several methods have been developed for the fabrication of these nanoantennas, including both top-down and bottom-up approaches [7]. The challenge of loading the interstice between the two arms with different materials, including single molecules [8], quantum dots [9], and nonlinear nanocrystals [10–12], has been taken up, enabling a strong control of light–matter interaction in these hybrid nanostructures.

At the same time, it was proposed to use nanoantennas for the observation of nonlinear optical processes at the nanoscale [13]. The basic idea in nonlinear plasmonics is to take advantage of the huge field enhancement associated with the excitation of LSPR in nanoantennas to obtain a high nonlinear conversation rate, despite very small interaction volumes (much smaller than μm^3^) [13]. This strategy has been proven to be very successful for various nonlinear optical processes, such as multiphoton photoluminescence [6], third harmonic generation [14–15], and four-wave mixing [16–17]. However, the enhancement of the nonlinear conversion rate for second harmonic generation (SHG) was found to be surprisingly low [18–19]. This is in contrast with the observations made for third order nonlinear optical processes. In that context, Berthelot et al. reported for the first time what they called the “silencing” of SHG from nanogaps, which could explain the low SHG observed experimentally [18]. These authors measured the SHG from the interstice between two gold nanowires with various gap sizes. They observed that the SHG does not increase as the gap between the two nanowires decreases, despite an increase of the fundamental field enhancement. Their experimental observations were supported by numerical results demonstrating that the SHG from nanogaps is not efficiently radiated out. Indeed, due to the specific selection rules of SHG [20], the second harmonic sources standing on each side of the nanogap are out of phase, i.e., point in opposite directions at a given time, and tend to cancel out in the far-field. The problem of the “silencing” of the SHG has also been addressed in the case of connected gold nanodimers by comparing the evolution of the SHG with that of the two-photon photoluminescence [21]. It was shown that, although these two nonlinear optical processes involved two fundamental photons, they did not have the same incident polarization dependence, revealing distinct behaviors as the fundamental near-field distribution changes. The role of the antenna modes at the second harmonic wavelength in the enhancement of the SHG has also been addressed for symmetric and asymmetric antennas, showing that the SHG is strongly modified by a gap displacement with respect to the antenna center [22]. In any case, the “silencing” of SHG from gold nanoantennas has not been addressed in detail so far. For example, the spatial origin of the second harmonic signal collected from plasmonic nanoantennas has not been clearly identified.

In this article, we report a comprehensive discussion of the SHG from gold dimers focusing on the implications of the silencing effect. The linear and second harmonic responses of the dimers have been computed using a surface integral equations method. The first dimers considered in this article are made of cylindrical nanorods with hemispherical extremities and various gap distances. In order to unveil the role of the silencing effect, computations of the SHG considering only the surface second harmonic sources on specific parts of the dimers are also presented. The meshes describing the dimers are then slightly deformed to mimic the presence of defects on the nanorod surfaces. Finally, gold dimers made of rectangular arms are considered.

## Numerical Methods

The linear optical response was calculated using a surface integral formulation [23–24]. All the nanostructures are embedded in a homogeneous medium with refractive index *n* = 1.33, corresponding to water. The dielectric constants of gold are taken from experimental data at both the fundamental and second harmonic wavelengths [25]. For the SHG computations, the linear surface currents are used for the evaluation of the fundamental electric fields just below the gold surfaces and then used for the calculation of the surface SH polarization [26–27]. Only the χ_surf,nnn_ component of the surface tensor (where *n* denotes the component normal to the surface) is considered. Recent experimental results show that this term dominates the surface response of metallic nanoparticles [28–29]. Note that other contributions to the SH signal, namely the χ_surf,ttn_ component of the surface tensor (where *t* denotes the component tangential to the surface) and bulk contribution, are theoretically allowed but these terms contribute only weakly to the total SH wave [28–29]. The SH surface currents are obtained by solving the surface integral equation formulation taking into account the nonlinear polarization and enforcing the boundary conditions at the nanostructure surfaces [30]. As the linear surface currents, the SH surface currents are expanded on Rao–Wilton–Glisson (RWG) basis functions. The expansion coefficients are found by applying the method of moments with Galerkin’s testing [23–24]. A Poggio–Miller–Chang–Harrington–Wu–Tsai formulation is used to ensure accurate solutions even at resonant conditions [23–24]. The SH electric field is then deduced from the SH surface currents [26–27].

## Results and Discussion

### Gold dimers made of cylindrical nanorods

**Figure 1:**
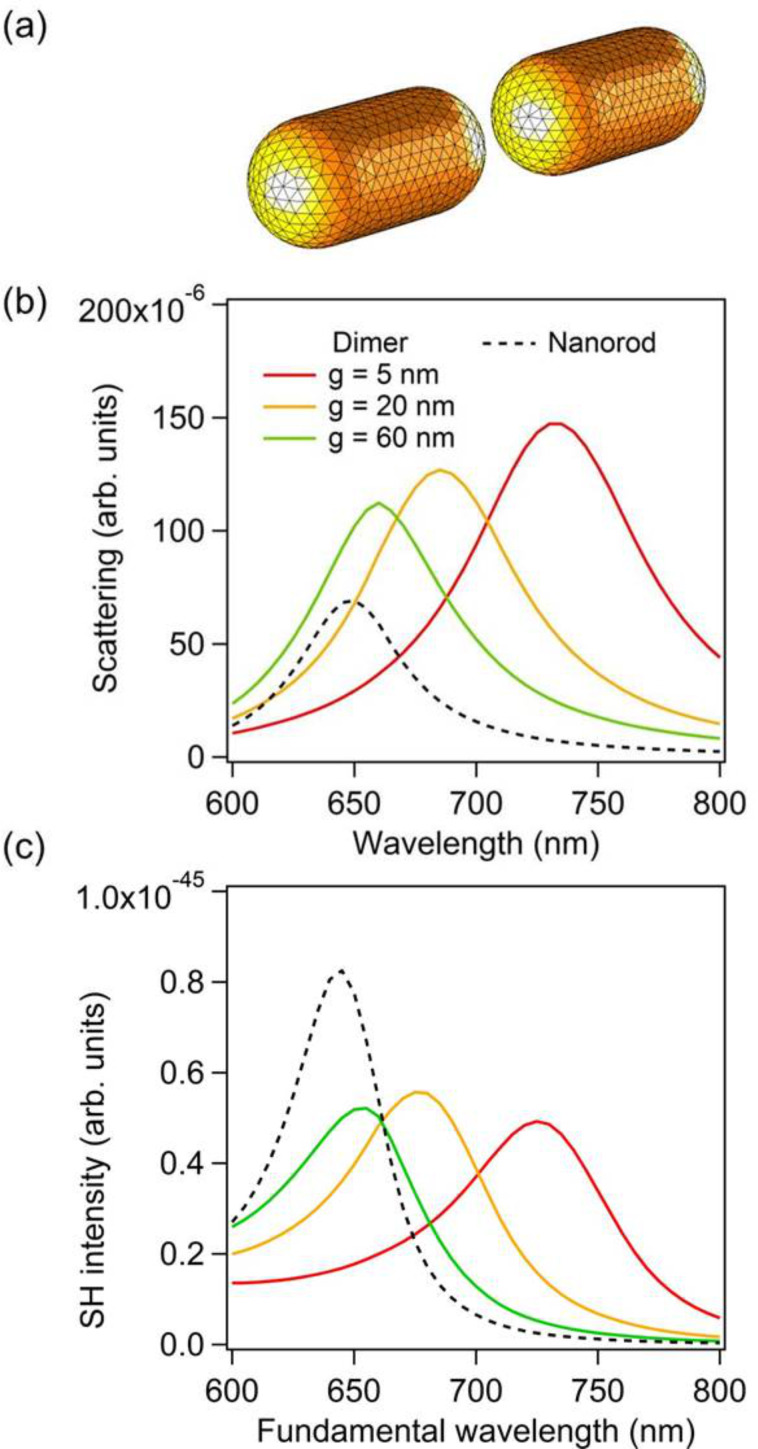
(a) Example of one mesh used for the simulations. The nanorod overall length and diameter are 85 nm and 40 nm, respectively. In the present case, the gap *g* is 20 nm. (b) Scattering intensity as a function of the wavelength for nanodimers with gaps *g* = 5 nm, 20 nm, and 60 nm as well as for a single nanorod. (c) Second harmonic intensity as a function of the wavelength for nanodimers with gaps *g* = 5 nm, 20 nm, and 60 nm as well as for a single nanorod.

We first consider the case of dimers made of gold nanorods, see Figure 1a. The diameter of the nanorods is 40 nm and their length is 85 nm. The smallest considered gap between the nanorods is 5 nm. Such small gaps can easily be achieved using capillary assembly with optimized templates, for example [7]. The linear optical properties of gold dimers with gaps of 5 nm, 20 nm, and 60 nm are considered first. The scattered intensity is shown as a function of the incident wavelength in Figure 1b. For comparison, the scattering from a single nanorod is also shown as a dashed line. In all the scattering spectra, one can observe a peak corresponding to the excitation of a LSPR. This LSPR corresponds to the bonding dipolar mode, resulting from the in-phase coupling of the longitudinal dipolar modes supported by each nanorod. As expected, this mode redshifts as the gap between the nanorods decreases, i.e., as the coupling increases [2]. As a consequence of this coupling, the bonding dipolar mode for a dimer always arises at a longer wavelength than the longitudinal electric dipole mode of the corresponding single nanorod. On the other hand, the scattering efficiency from the single nanorod is lower than that of the considered dimers, as shown in Figure 1b.

**Figure 2:**
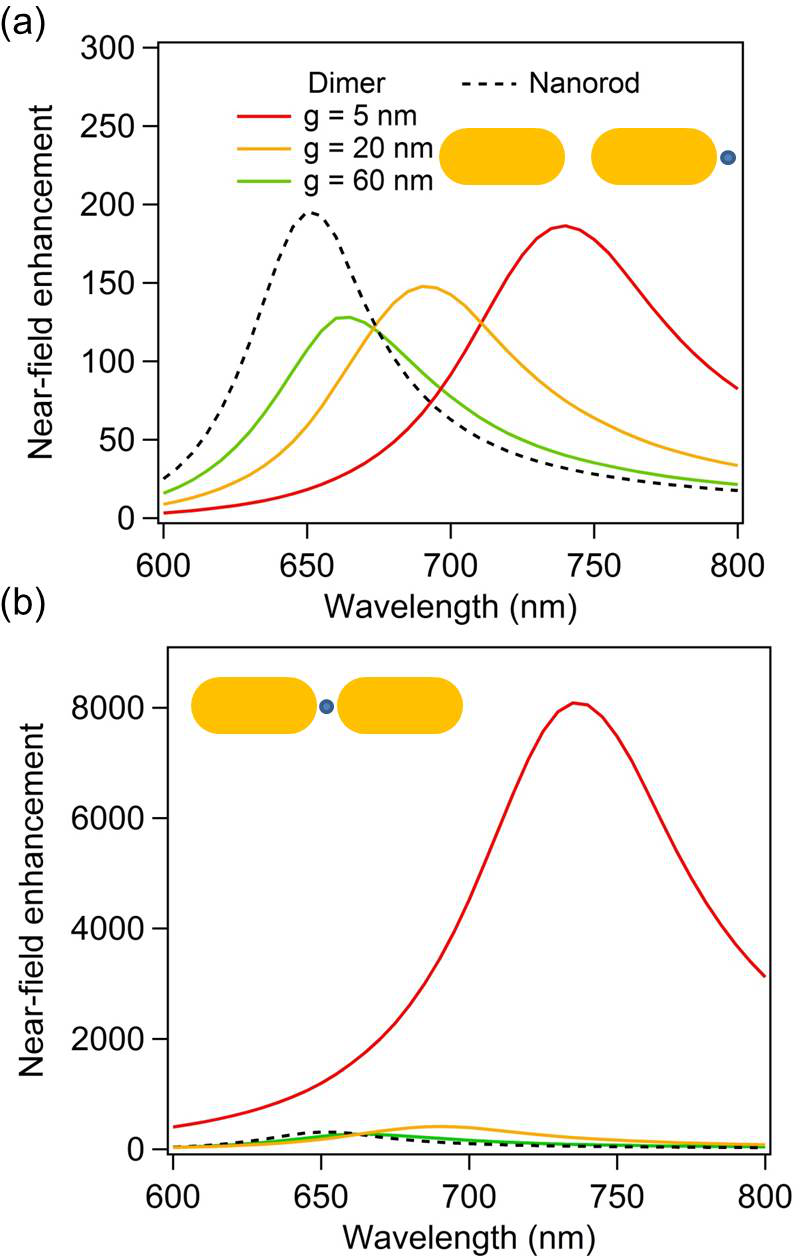
(a) Enhancement of the fundamental intensity evaluated at the nanorod extremities (2.5 nm away from the surface) as a function of the wavelength for nanodimers with gaps *g* = 5 nm, 20 nm, and 60 nm as well as for a single nanorod. (b) Enhancement of the fundamental intensity evaluated at the gap center as a function of the wavelength for nanodimers with gaps *g* = 5 nm, 20 nm, and 60 nm as well as for a single nanorod. The field enhancement is evaluated at 2.5 nm away from the nearest surface.

We now turn our attention to the SHG from the same gold dimers and a single nanorod. Figure 2c shows the far-field second harmonic intensity as a function of the fundamental wavelength for gold dimers with gaps of 5 nm, 20 nm, and 60 nm and a single nanorod. For each nanostructure, a maximum of the second harmonic scattering is observed when the fundamental wavelength is close to the resonant wavelength of the bonding dipolar mode. This effect is well-known and has been reported in various publications – it has been established, as a cornerstone of nonlinear plasmonics, that the nonlinear optical responses of plasmonic nanostructures is boosted by LSPRs [31–32]. Indeed, a strong near-field enhancement is associated with the collective oscillations of the conduction electrons, resulting in an increased nonlinear polarization and then in an increase of the nonlinear optical conversion for example. It is however apparent in Figure 1c that the maximum of SHG is not directly related to the gap size as one would expect. To understand and explain this phenomenon, the near-field intensity enhancement is now discussed.

The fundamental intensity enhancements for the gold dimers have been evaluated at the dimer extremities, respectively in the nanogap, and are shown in Figure 2 as a function of the fundamental wavelength, respectively in panels (a) and (b). In order to track the SH sources at the surface of the different nanostructures, the intensity enhancement is always evaluated 2.5 nm away from the nearest surface, i.e., not at the center of the gaps for *g* = 20 nm and 60 nm. In this context, it is worth noting that the fundamental electric field used to compute the SH sources is directly evaluated from the linear surface currents. Furthermore, the boundary conditions of the electromagnetic field are linear and a stronger fundamental electric field in the gap leads to stronger SH sources in this area. At both the nanodimer extremities and the nanogap, the field enhancement increases as the gap between the nanorods decreases, although the evolution of the intensity enhancement is more dramatic in the gap (note the different scales in Figure 2a,b). Indeed, the intensity enhancement in the gap increases from ≈300 for a gap of 60 nm to ≈8000 for a gap *g* = 5 nm. From this last observation, one can naively think that the SHG would be much higher for the smallest gap, due to the strong near-field intensity. However, this is not the behavior observed in Figure 1b. Indeed, the far-field SH intensity tends to increase with the gap between the nanorods. This effect is known as the “silencing” of the SHG, meaning that the far-field SH intensity decreases despite an increase of the fundamental near-field intensity.

**Figure 3:**
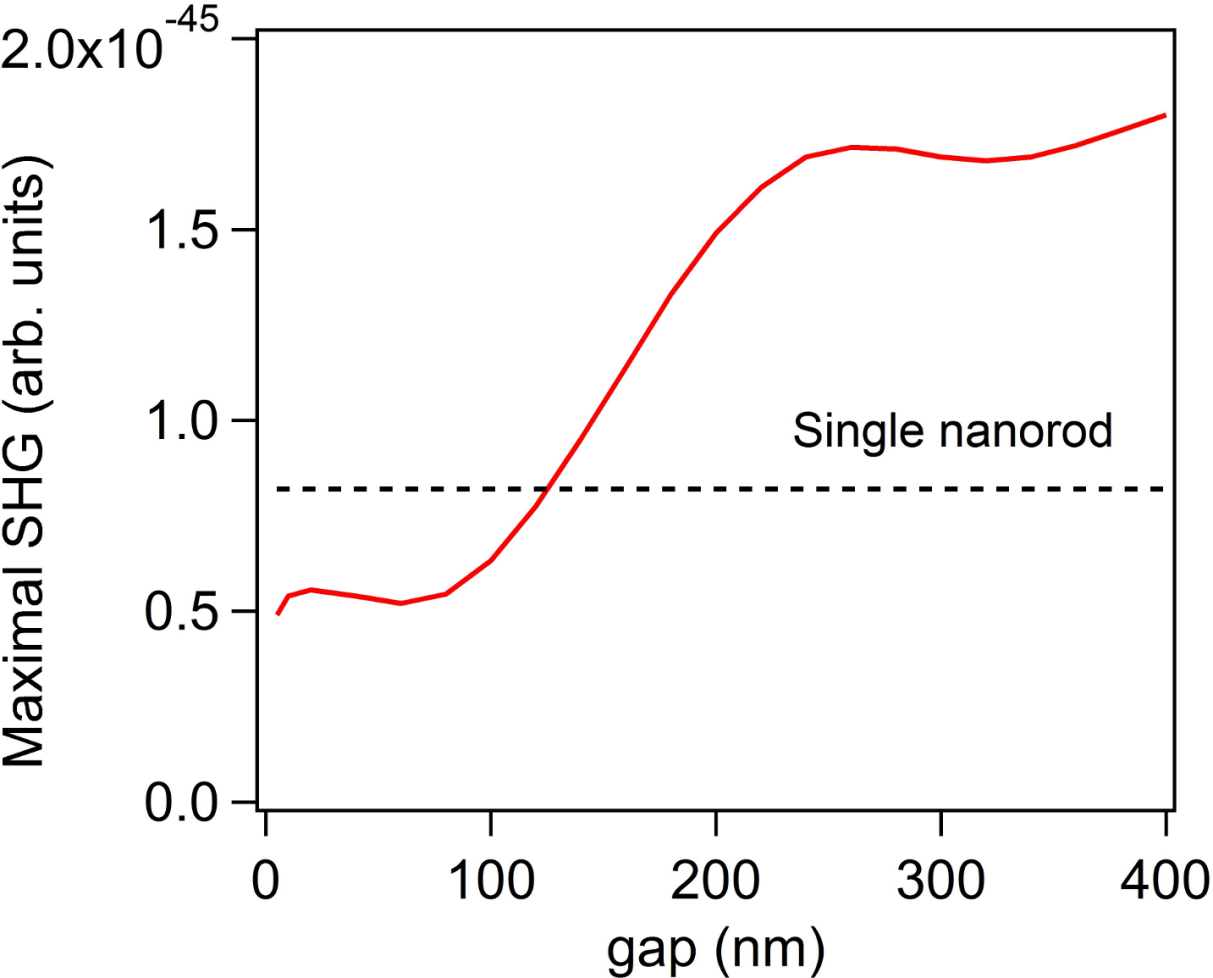
Maximal second harmonic generation (SHG) as a function of the gap between the nanorods. The maximal SHG has been extracted from the second harmonic spectra, see Figure 1c for example. The SH intensity from a single nanorod is shown as a dotted black line.

This particular behavior is explained by the specific symmetry properties of SHG. The second harmonic sources standing at each side of the nanogaps are out of phase, i.e., pointing towards each other, resulting in a vanishing SHG in the electric dipole approximation. As a consequence, the far-field SH radiation decreases when the distance between the SH sources shrinks. While this is the standard interpretation of the silencing effect, there are several points that need to be clarified. The first one is the role of the resonant wavelength of the bonding dipolar mode. The SHG from centrosymmetric nanostructures is induced by retardation effects, i.e., the field variation across the structure both at the excitation and emission stage, which increases as the fundamental and SH wavelengths decrease [33–34]. For this reason, the redshift of the bonding dipolar mode observed for small gaps between the nanorods is not beneficial for a high SHG. Please note that for the range of SH wavelengths considered in this study, the imaginary part of the dielectric constant of gold is constant and does not modify the losses at the SH wavelength. Figure 3 shows the maximal SHG induced by the resonant excitation of the bonding dipolar mode at the fundamental wavelength for gaps ranging from 5 nm to 400 nm. For the largest gaps, the bonding dipolar mode is spectrally close to the longitudinal mode of the single nanorod and does not shift with gap size variations. Thus, in the large gap case, the variation of the SHG is due to interference of the fields radiated by each nanorod, which simply changes with the distance between the arms since the LSPR does not shift. Quite surprisingly, the highest SHG is obtained for the largest gaps, despite a weak enhancement of the fundamental intensity in the gap between the nanorods. The far-field second harmonic intensity is identical to that of the single nanorod for a gap of 130 nm and is more than twice that of the single nanorod for gaps larger than 220 nm, see Figure 3. Note that, if the local field enhancement is negligible (equal to unity), then the highest possible SHG from a dimer is 4 times that of the single nanorod, since SHG is a coherent optical process. The slight modulation of the second harmonic intensity observed for gaps larger than 220 nm is attributed to the radiative coupling between the nanorods, which slightly modifies the fundamental near-field enhancement as the distance between the nanorods varies. Having discussed the role of the resonant wavelength with respect to the silencing of the SHG, we now turn our attention to the second important point: the position of the second harmonic sources and their contribution to the overall far-field SH signal.

### Decomposition of the second harmonic sources

**Figure 4:**
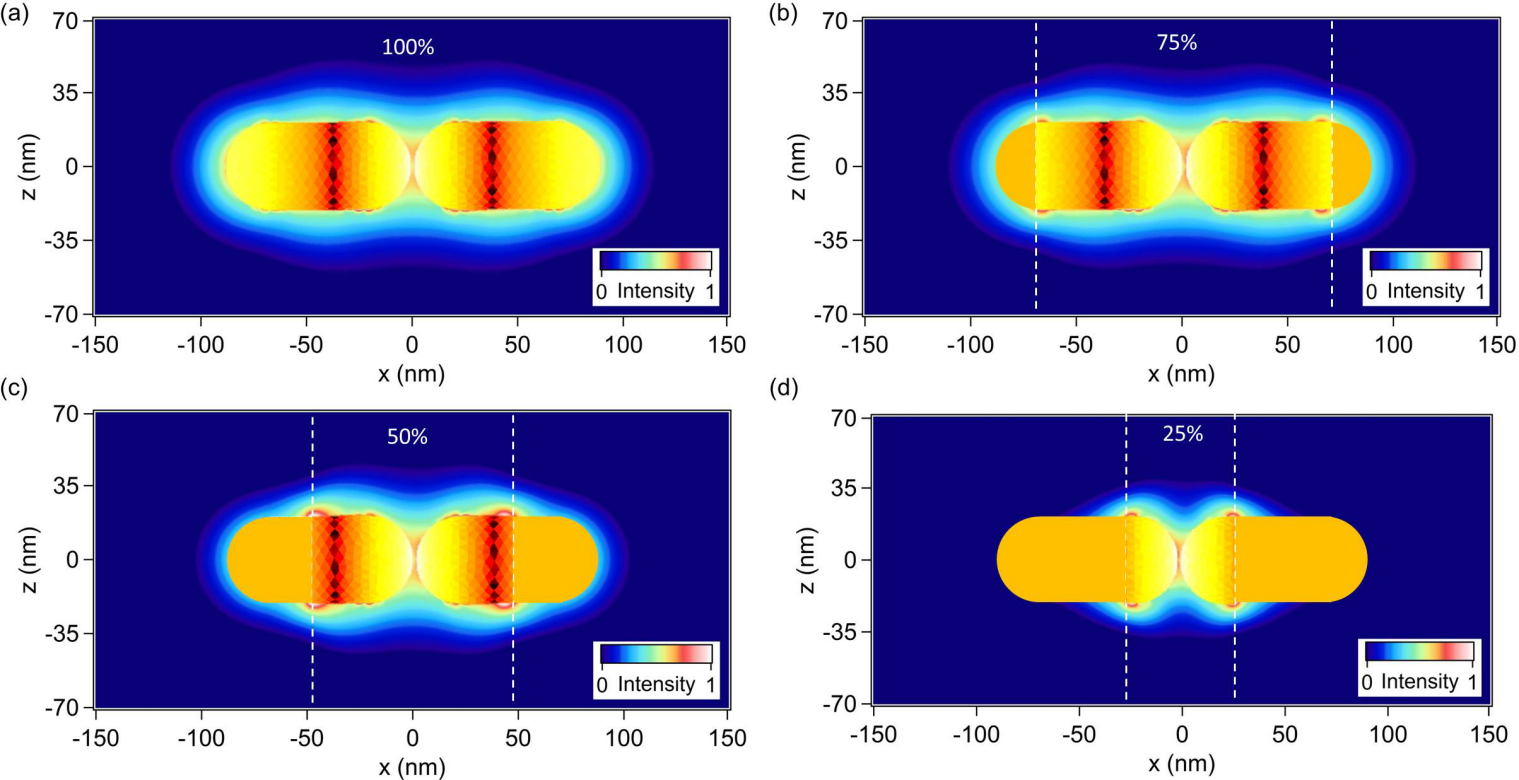
Normalized near-field distributions of the second harmonic electric field intensity close to the gold nanodimer with a gap *g* = 5 nm evaluated (a) for the entire surface nonlinear polarization and for (b–d) partial surface nonlinear polarizations. The percentages indicate the ratio between the length over which the surface nonlinear polarization is maintained and the total length of the nanorod. For example, 50% indicates that only half of the surface nonlinear polarization is considered as shown in panel (c). The same color scale is used for all the panels.

**Figure 5:**
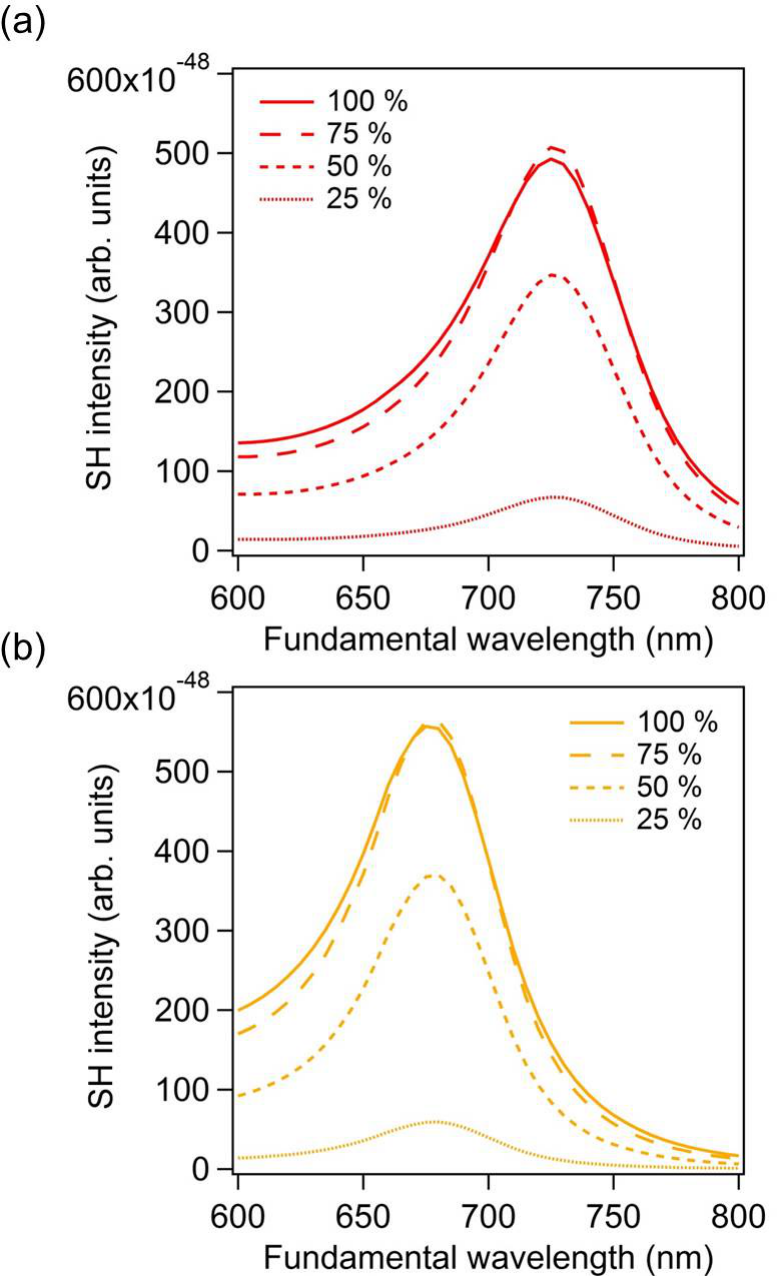
Far-field second harmonic intensity for the entire surface nonlinear polarization (100%) and for partial surface nonlinear polarizations. The percentages indicate the ratio between the length over which the surface nonlinear polarization does not vanish and the length of the nanorod, see Figure 4. The gap between the nanorods is 5 nm for the panel (a) and 20 nm for panel (b).

In this section, the important question of the relationship between the location of the second harmonic sources and their contributions to the overall far-field SH intensity is addressed. Indeed, the second harmonic sources are distributed over the entire gold nanodimer surface. It is clear that the second harmonic sources, i.e., the second harmonic surface polarization, are the strongest in the nanogaps, due to the huge fundamental field-enhancement in those locations. However, as discussed previously, these sources do not radiate efficiently into the far-field due to the silencing of the SHG and the link between the SH source locations and their contribution to the collected SH signal is still an open question. To address this point, we perform computations of the SHG from the gold dimers, limiting the surface covered with second harmonic sources. Note that the entire nanostructure is considered for the computations, meaning that the modal distribution is conserved at the SH wavelength, and only portions of the nonlinear surface polarization are suppressed, see Figure 4. Thus, the partial nonlinear sources still induce nonlinear currents over the entire structure, which can then scatter the SH field. Furthermore, to preserve the symmetry of the problem, the nonlinear surface polarization is identically suppressed for both arms. The percentages in Figure 4 indicate the length over which the nonlinear surface polarization is maintained. Note that the central region of the nanodimer always has a non-vanishing surface SH polarization. The far-field SH intensity has been evaluated for various partial surface nonlinear polarizations (25%, 50%, 75%, and 100%) for a gap of 5 nm, Figure 5a, and a gap of 20 nm, Figure 5b. Removing the nonlinear polarization at the extremities of the nanorods increases slightly (*g* = 5 nm) or does not change (*g* = 20 nm) the SH emission, meaning that these SH sources do not really contribute to the far-field emission and can even interfere destructively with other sources. Considering the SH sources in the nanogap only (coverage of 25%), the far-field SH intensity drops by a factor 7 for a gap *g* = 5 nm and by a factor 10 for a gap *g* = 20 nm in comparison with the entire nonlinear polarization, emphasizing again the weak emission from this area, especially for small gaps. On the basis of these observations, one can conclude that the second harmonic sources on the side of the nanorods give an important contribution to the second harmonic radiation. At this point, it is worth reminding that the SHG from centrosymmetric nanostructures is induced by retardation effects. The phase variation of the incident wave induces one channel for the second harmonic emission, which corresponds to a second harmonic dipole along the propagation direction of the pump wave.

### Influence of surface defects on the SHG

**Figure 6:**
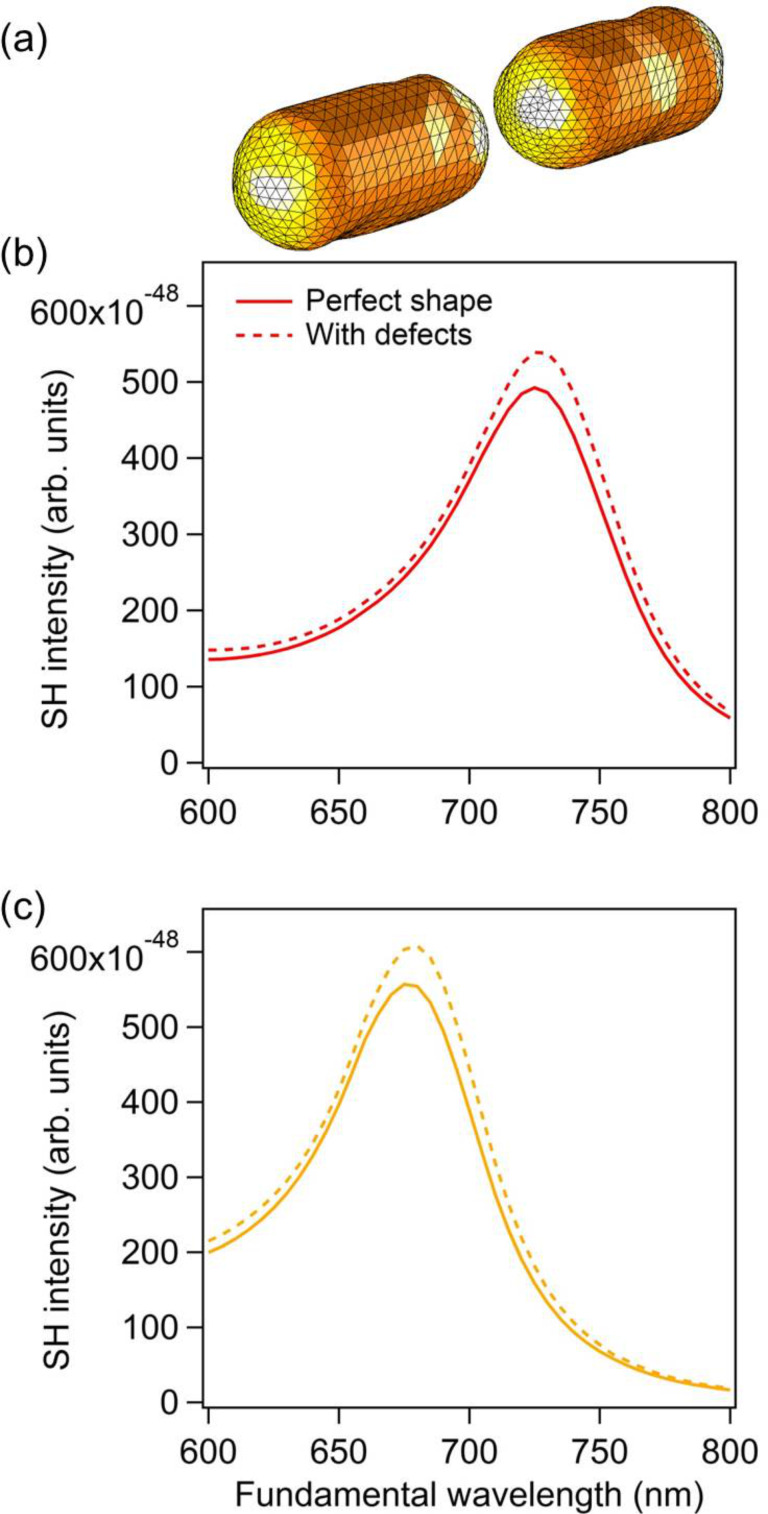
(a) Mesh used for the dimers with defects. In this example, the gap is 20 nm. Far-field second harmonic intensity for a dimer made of perfect nanorods (solid lines) and with defects (dashed lines). The gap between the nanorods is 5 nm for panel (b) and 20 nm for panel (c).

SHG from centrosymmetric nanostructures is forbidden in the electric dipole approximation and one can expect that SHG would be sensitive to any centrosymmetry breaking [35–36]. As discussed in the previous sections, the centrosymmetry can be indeed broken by the retardation of the incident field, resulting in the excitation of a second harmonic dipole along the propagation direction of the incident planewave for example [33–34]. The second possibility is a centrosymmetry breaking induced by the nanoparticle shape [35–36]. Indeed, although important progress has been made in nanofabrication, it still impossible to fabricate nanostructures of perfect shape and, for real experiments, one must consider the influence of shape variations of the nanostructures on SHG. This issue has been thoroughly considered in the case of SHG from chemically synthetized plasmonic nanospheres in solution, revealing an interesting competition between the centrosymmetry breaking induced by the field variation and the nanoparticle shape [35,37–39]. To investigate this issue [40], the meshes describing the nanodimers have been modified as follows. A few points over the mesh are first randomly selected. Each of these points and their nearest neighbors are then smoothly moved towards the interior of the structure, while the deformation is maximal at the selected point and vanishes a few nanometers away. As expected, the far-field second harmonic intensity increases due to the defects on the nanorod surfaces, see Figure 6. However, the second harmonic intensity increase is relatively modest, only 10%, meaning that the SHG is mainly due to the retardation effects for this range of nanorod sizes and deformations. This observation stands for the overall second harmonic emission, integrated over a sphere. Nonetheless, the centrosymmetry breaking induced by the defects modifies the selection rules. As a consequence, the second harmonic intensity does not vanish anymore in the forward and backward directions. This is an important observation from a practical point of view, since the second harmonic light is often collected in these two directions. Note that the case of a single defect located in the gap has been considered in a previous publication, showing the same behavior [36].

### Gold dimers made of rectangular arms

**Figure 7:**
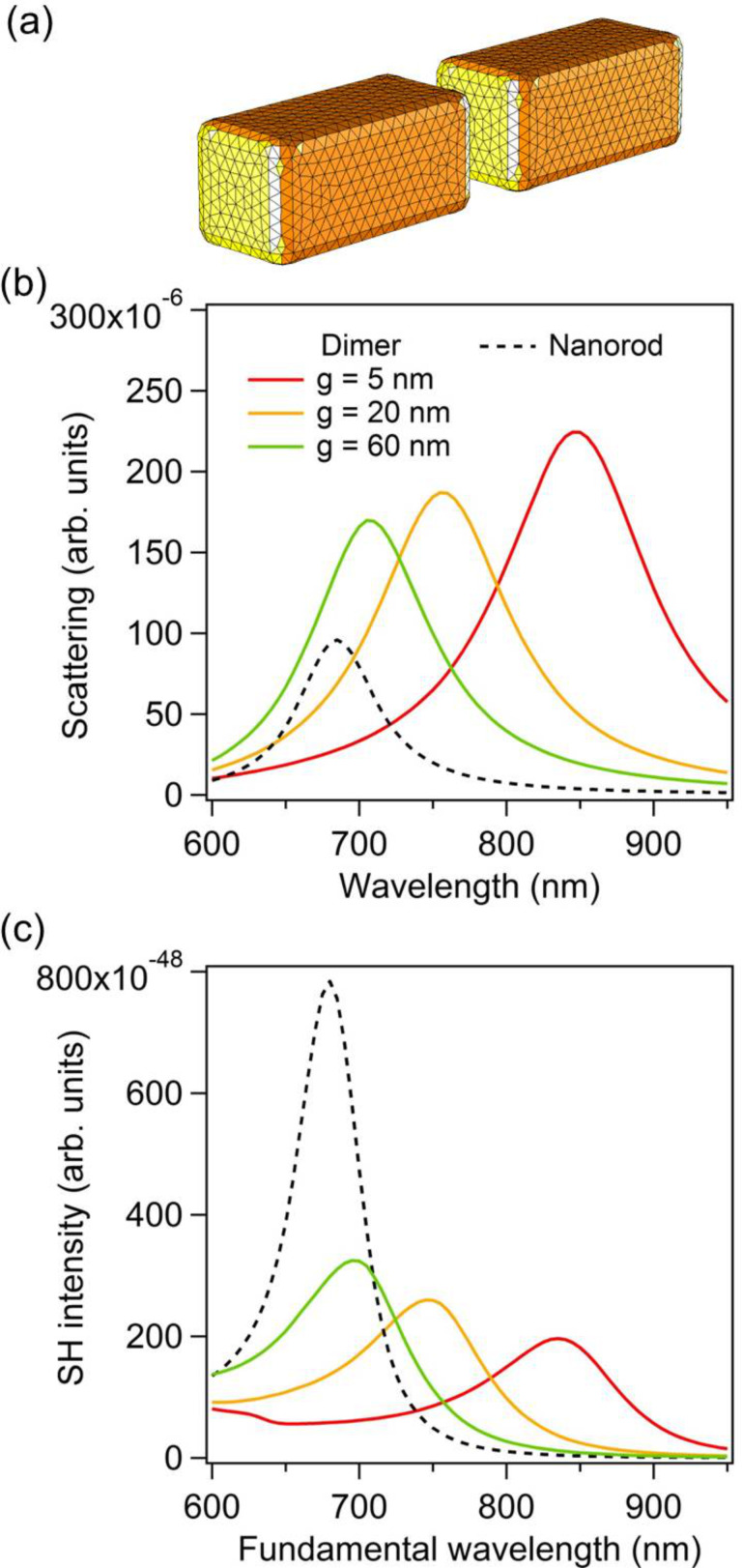
(a) Example of one mesh used for the simulations for the dimers with rectangular arms. The nanoparticle length is 85 nm and the height and width are 40 nm. In the present case, the gap *g* is 20 nm. (b) Scattering as a function of the wavelength for nanodimers with gaps *g* = 5 nm, 20 nm, and 60 nm as well as for a single nanoparticle. (c) Second harmonic intensity as a function of the wavelength for nanodimers with gaps *g* = 5 nm, 20 nm, and 60 nm as well as for a single nanorod.

**Figure 8:**
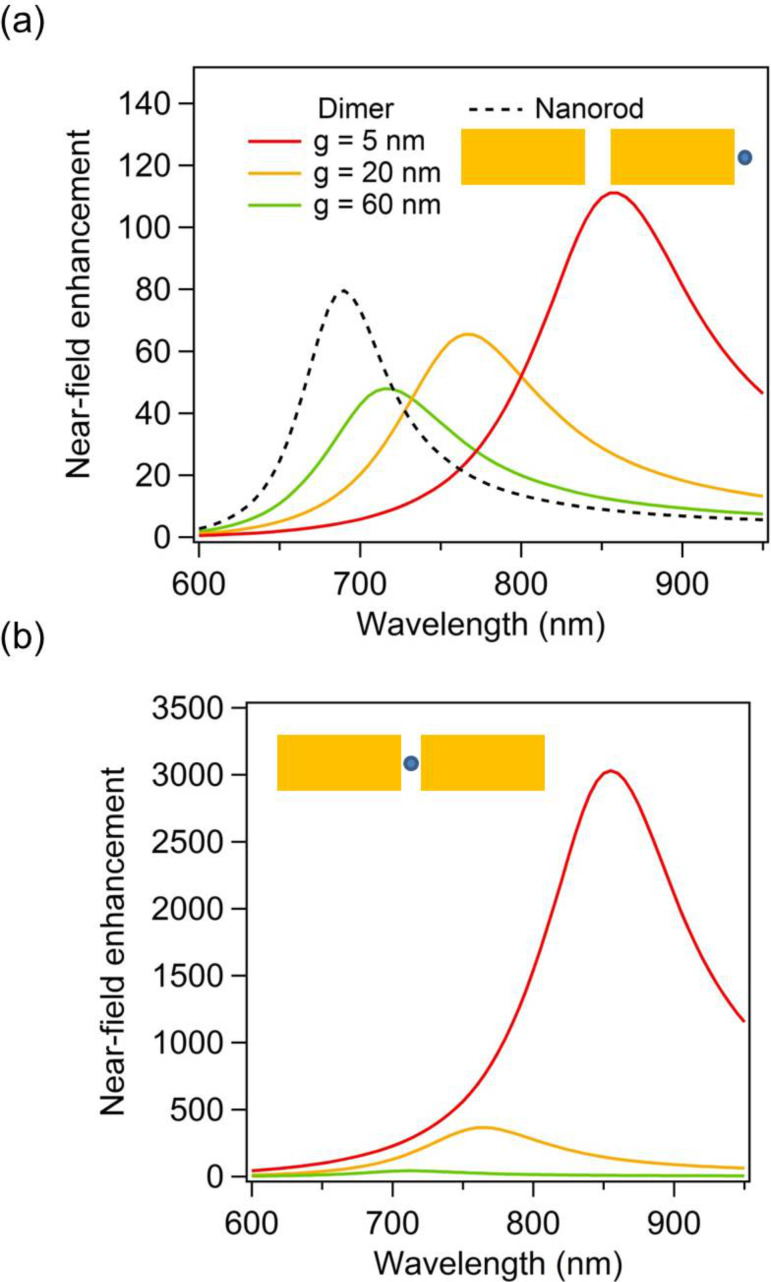
(a) Enhancement of the fundamental intensity evaluated at the nanorod extremities (2.5 nm away from the surface) as a function of the wavelength for nanodimers with gaps *g* = 5 nm, 20 nm, and 60 nm as well as for a single nanorod. (b) Enhancement of the fundamental intensity evaluated at the gap center as a function of the wavelength for nanodimers with gaps *g* = 5 nm, 20 nm, and 60 nm as well as for a single nanorod. The field enhancement is evaluated at 2.5 nm away from the nearest surface.

**Figure 9:**
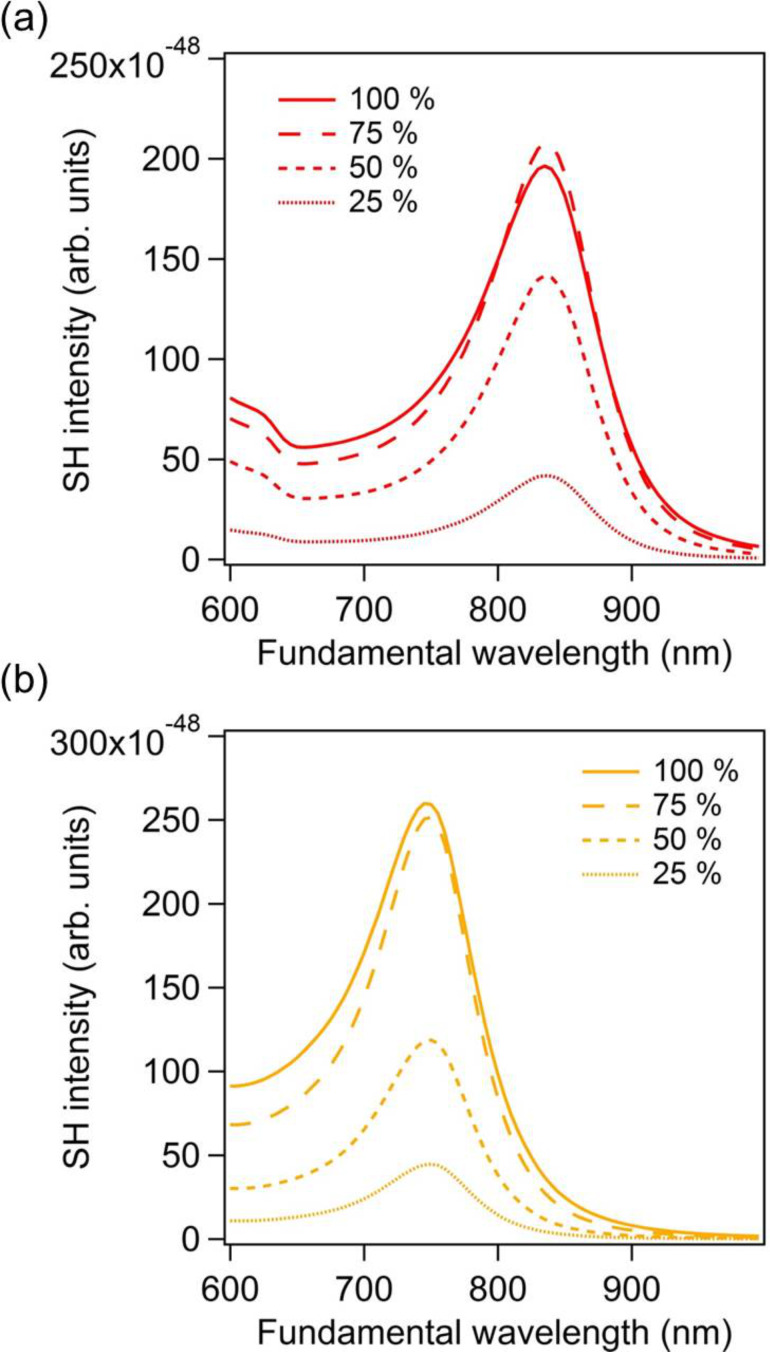
Far-field second harmonic intensity for the entire surface nonlinear polarization (100%) and for partial surface nonlinear polarizations as indicated in the legends. The percentages indicate the ratio between the length over which the surface nonlinear polarization is maintained and the length of the nanorod, as in Figure 4 for the nanodimers with cylindrical sections. The gap between the nanorods is 5 nm for the panel (a) and 20 nm for panel (b).

In this last section, the case of gold dimers made of rectangular arms is considered, Figure 7a. The purpose is to show that the observations made for cylindrical nanorods still stand for other geometries. Both geometries can be obtained with specific nanofabrication techniques. While cylindrical nanorods would be the building blocks of plasmonic nanoantennas made by capillary assembly, rectangular arms will be obtained with top-down fabrication techniques, such as nanolithography for example. The arm dimensions are similar to those of the cylindrical nanorods discussed previously; the arm length is 85 nm and its width and height are 40 nm. The scattering spectra for such gold dimers with gaps of 5 nm, 20 nm, and 60 nm are shown in Figure 7b, revealing a redshift of the LSPR as the gap between the arms decreases as expected. This redshift of the LSPR decreases the retardation effects, and then the maximal SHG, as the gap between both arms decreases, Figure 7c. Contrary to the cylindrical nanorods, the SHG continuously decreases while the gap increases from 5 nm to 60 nm. This difference is probably due to the gap geometry. Indeed, with rectangular arms, the near-field coupling is much stronger since flat surfaces result in higher charge interaction in the nanogap, and then in a larger LSPR shift. For the same gap variation, i.e., from 5 nm to 60 nm, the shift of the LSPR for the rectangular arms is twice that observed for cylindrical nanorods. Apart from the plasmon shift amplitudes, the near-field behavior is similar for the rectangular arms, i.e., a strong near-field enhancement is observed in the gap for the smallest gaps (Figure 8), meaning that the SHG from this kind of nanoantennas is also ruled by the “silencing effect”. Finally, the original approach proposed in this article is applied to the nanoantennas with rectangular arms. Figure 9 shows the far-field second harmonic intensity considering all or some parts of the nonlinear surface polarization for gaps of 5 nm and 20 nm. As for the cylindrical nanorods, the results indicate that the second harmonic sources on the sides of the arms play a non-negligible role in the total second harmonic radiation. To summarize the results of this section, one can note that the geometry of the arms does not play a significant role, beyond the resonant wavelength, in the “silencing” of the SHG, which is intrinsic to the geometry of nanogaps. Of course, the “silencing” of the SHG can be reduced by designing non-centrosymmetric gaps, as the one observed in T-shaped nanostructures [41], V-shaped nanoantennas coupled to nanorods [42], and nanorod–nanodisk systems [43].

## Conclusion

In conclusion, this article reports a comprehensive study of the “silencing” effect in the SHG from gold nanoantennas using a surface integral equation method. To investigate this phenomenon in detail, various geometries have been considered, including nanoantennas with cylindrical and rectangular arms, as well as surface defects. To quantify the “silencing” effect, a new numerical approach, in which only specific parts of the nonlinear surface polarization are considered, has been developed. The results show that only a small part of the overall SH emission indeed comes from the nanogaps, as a consequence of the “silencing” effect. The numerical results reported in this article clearly demonstrate that the SH sources located away from the gap, especially those on the arm sides, play a significant role in the overall SH emission. This point had not yet been discussed in the literature, probably because the “silencing” effect had first been reported for nanogaps between long arms [18], the properties of which are different from those of typical nanoantennas, resulting in different SH source distributions. The present discussion provides new directions for the design of efficient nanosources of SH light and meta-atoms for the fabrication of nonlinear metasurfaces [44–46], especially regarding the role of the SH source distribution over nanostructure surfaces. On the other hand, SHG was used to monitor the gap distance between gold nanoparticles and gold films [47]. The present study underlines the fact that the relationship between SHG and field enhancement in nanogaps is not straightforward and nonlinear plasmonic nanorulers need to be carefully calibrated [48]. Finally, it would be interesting to extend the present study to aluminum nanoantennas, since a significant bulk quadrupolar SHG is expected in this case [49].
